# A first report of *Pseudosuccinea columella* (Say, 1817), an alien intermediate host for liver fluke, in Malawi

**DOI:** 10.1186/s13071-024-06241-5

**Published:** 2024-04-11

**Authors:** S. Jones, A. Juhász, P. Makaula, L. J. Cunningham, J. Archer, C. Nkolokosa, G. Namacha, E. Kambewa, D. Lally, D. R. Kapira, P. Chammudzi, S. A. Kayuni, J. Musaya, J. Russell Stothard

**Affiliations:** 1 Department of Tropical Disease Biology, Liverpool School of Tropical Medicine, Liverpool, L3 5QA UK; 2 Semmelweis University, Budapest, Hungary; 3 Malawi-Liverpool-Wellcome (MLW) Clinical Research Programme, Blantyre, Malawi

**Keywords:** *Fasciola*, Lake Malawi, Lymnaeids, Molecular xenomonitoring, Shire River Valley

## Abstract

Starting in October 2021, quarterly malacological surveys have been undertaken in Malawi, with the sampling of 12 specified freshwater habitats throughout a calendar year. Each survey monitors the presence of aquatic intermediate snail hosts of medical and veterinary importance. In March 2023, the alien lymnaeid species *Pseudosuccinea columella* was encountered for the first time in the surveys, in Nsanje District. This species identity was later confirmed upon DNA analysis of mitochondrial ribosomal 16S sequences. In July 2023, *P. columella* was also noted at single sites within Mangochi and Chikwawa Districts, and again in Nsanje District, with an additional location observed. Of particular importance, our sampled location in Mangochi District was directly connected to Lake Malawi, which expands the species list of invasive molluscs in this lake. While *P. columella* is a well-known intermediate snail host for human and animal fascioliasis, screening collected snails for trematode cercariae, alongside molecular xenomonitoring, did not yield equivocal evidence of active fluke infection. However, the newly recognized presence of this alien intermediate snail host within Lake Malawi, and along the Shire River Valley, flags a new concern in altered local transmission potential for human and animal fascioliasis.

**Graphical Abstract:**

**Figure:**
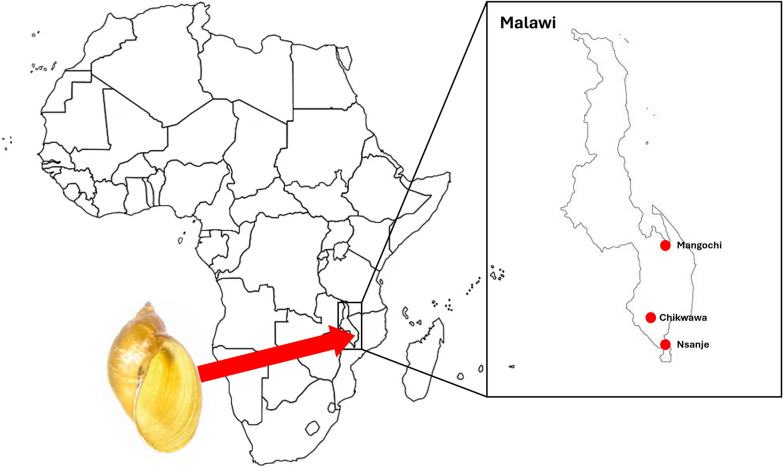

*Pseudosuccinea columella* (Say, 1817), an invasive snail species originating from the Americas, has successfully colonized many freshwater habitats in Africa [1]. In southern Africa, its dispersion is recorded in Namibia, South Africa and Zimbabwe [2–5]. As *P. columella* is an alien intermediate host of liver fluke, its distribution is of medical and veterinary interest.

Our unexpected encounter with *P. columella* arose from broader malacological surveys for intermediate snail hosts of schistosomiasis, as part of activities performed within the framework of the “Hybridisation in UroGenital Schistosomiasis (HUGS)” project. Commencing in October 2021, then at quarterly intervals, HUGS has been inspecting 12 locations using standard freshwater snail collection protocols in Mangochi District (*n* = 7 sampling locations), Chikwawa District (*n* = 2) and Nsanje District (*n* = 3) (Fig. 1). The chosen locations are exemplars of high-risk water contact sites for human and animal schistosomiasis, and each location was inspected by a team of three, with snails collected by hand or by metal scoop depending on the habitat type [6].

**Fig. 1:**
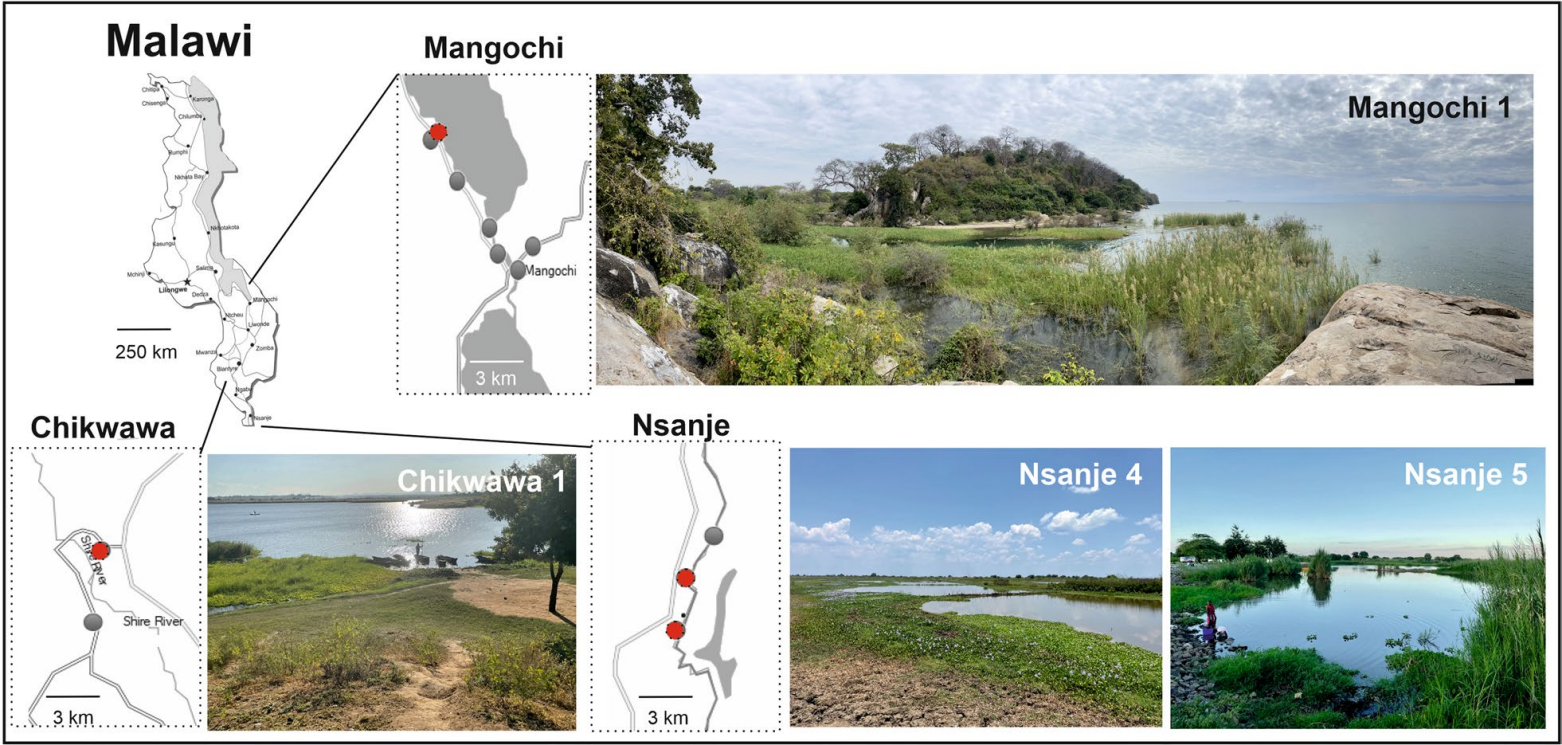
Sketch maps of the distribution of *Pseudosuccinea columella* in Mangochi (**a**), Chikwawa (**b**) and Nsanje (**c**) Districts, southern Malawi. Red circles indicate HUGS survey sites where *P. columella* was found; grey circles are surveyed sites where this snail was not found. The locations are: Mangochi 1 (− 14.31373°, 35.14174°); Chikwawa 1 (− 16.03759°, 34.84091°); Nsanje 4 (− 16.88780°, 35.27475°); Nsanje 5 (− 16.92985°, 35.26552°) with corresponding location photograph. Note that the panorama image of Mangochi 1 clearly shows the stream, flowing left to right, directly connected to Lake Malawi. HUGS, Hybridisation in UroGenital Schistosomiasis (project)

Owing to its distinctive shell micro-sculpture [7], which permits quick in-field differentiation from *Radix natalensis* (Krauss, 1848),* P. columella* was first noticed during the March 2023 survey. Thereafter, a more purposeful search for living snails was made during the July 2023 survey. In so doing, sufficient specimens (*n* = 6) were obtained for an anatomical inspection, molecular snail taxonomy investigation and molecular liver fluke xenomonitoring investigation, with additional specimens (*n* = 29) checked for shedding liver fluke cercariae. Several specimens of *R. natalensis* were collected concurrently for later comparison.

For the anatomical investigation, each snail was placed in water for 2 min at 80 °C. Soft tissues were then carefully removed from the shell with forceps, and the empty shell viewed under a dissecting microscope and photographed (Fig. 2a, b, e, f), alongside more detailed inspection of the shell’s periostracum (Fig. 2c, g). To view the radula, head tissue was first separated and incubated in lactic acid for 3 days; the radula was then mounted onto a glass slide, with glass coverslip overlaid, and photographed under a light microscope (magnification: ×1000). Particular attention was given to the morphology of the central and first lateral teeth (Fig. 2d, h).

**Fig. 2:**
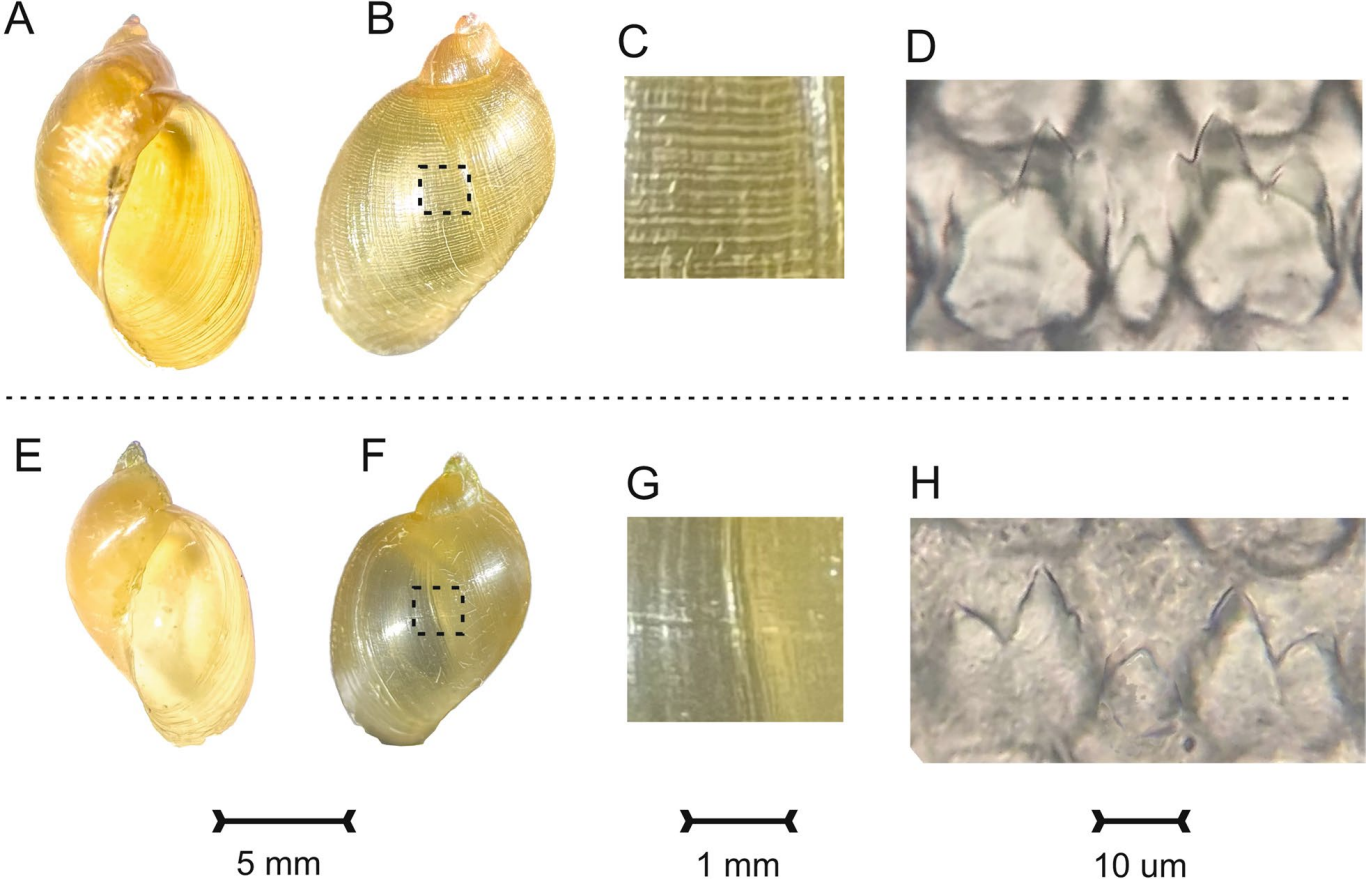
Conchological and anatomical comparison of *Pseudosuccinea columella* (top row) and *Radix natalensis* (bottom row).** a**–**d** *P. columella* conchology (**a**, **b**), shell microsculpture of the black square hatched area (**c**) and radular teeth (**d**)** e**–**h** *R. natalensis* conchology (**e**, **f**), shell microsculpture of the black square hatched area (**g**) and radular teeth (**h**). Although there is minor variation in the shape of the inner cusp of the first lateral teeth, the discriminatory feature is the periostracum’s spiral ridges

For the molecular taxonomy study, snail genomic DNA was extracted using the cetyltrimethylammonium bromide (CTAB) method, as adapted from [8]. Prior to tissue lysis, Phocine Herpes Virus (PhHV) was added to snail tissues as an internal extraction and later PCR amplification control for molecular xenomonitoring. Extracted genomic DNA was quantified using a NanoDrop spectrophotometer (Thermo Fisher Scientific, Waltham, MA, USA) then normalized to 10 ng/ul using ddH_2_O. In this study, we targeted a partial region of the mitochondrial ribosomal 16S gene using the universal primers 16brm (5′-CCGGTCTGAACTCTGATCAT-3′) and 16arm (5′-CGCCTGTTTATCAAAAACAT-3′) for PCR amplification, as described by Remigio [9]. After amplification, the purity of the amplicons was determined by agarose gel (2%) electrophoresis with SYBR™ Safe DNA Gel Stain (Invitrogen, Thermo Fisher Scientific) before Sanger sequencing with forward and reverse primers at Source BioScience (Source BioScience, Cambridge, UK). Upon analysis of the forward and reverse sequences with MEGA11 software [10], a single ribosomal 16S consensus sequence of 402 nucleotides in length was obtained and then submitted to a BLAST search [11]. Newly obtained sequences were then deposited in GenBank (accession number OR801605).

To perform molecular xenomonitoring of liver fluke infection, we used the TaqMan real-time PCR-based assay of Alasaad et al. [12], using the genus-specific primers SSCPFaF (5′-TTGGTACTCAGTTGTCAGTGTG-3′) and SSCPFaR (5′-AGCATCAGACACATGACCAAG-3′) with species-specific TaqMan probes to detect *Fasciola hepatica* (Linnaeus, 1758) (ProFh: 5′-[6FAM]ACCAGGCACGTTCCGTCACTGTCACTTT[BHQ1]-3′) and *Fasciola gigantica* (Cobbold, 1856) (ProFg: 5′-[HEX]ACCAGGCACGTTCCGTTACTGTTACTTTGTC[BHQ1]-3′). Real-time PCR reactions were performed using a MIC thermocycler (Bio Molecular Systems, Upper Coomera, Queensland, Australia) with genomic DNA from adult worms of *F. gigantica* originating from cattle in Uganda as the positive control.

Our BLAST search identified a sequence with 100% similarity: *P. columella* isolate LS3 mitochondrion genome (accession number NC_042905.1) from North America. Twenty additional identical 16S matches were noted, including *P. columella* from Brazil [13] and South Africa [14]. While we did not observe any snails shedding fluke cercariae, we did note, from molecular xenomonitoring, very weak amplification DNA signatures, with cycle threshold (Ct) values of 35. We consider these to most likely arise from spurious amplification of other trematode larvae [15]. In Africa, human and animal liver fluke is typically transmitted by freshwater snails of the genus *Galba* or *Radix* [16], giving rise to an often allopatric transmission of *F. hepatica* and *F. gigantica*, respectively [17]. Given the ability of *P. columella* to transmit both species of liver fluke [18], this alien intermediate host snail potentially adds a new dimension to this snail-parasite relationship in Malawi, although our current conclusion is that there was no evidence for active liver fluke infection within our sampled snails.

To provide an insight into the ecology of *P. columella*, we review here, in brief, the aquatic habitats where it was found. As shown in Fig. 1, sampling site Mangochi 1 is predominantly a stream habitat, immediately marginal and directly connected to Lake Malawi itself. Since Lake Malawi is well-known internationally as a global hotspot of biological diversity, the addition of *P. columella* to its species list is not trivial in the least. Indeed, its presence likely adds to the expanding list of ecological change within the lake and is pertinent to other snail-borne diseases locally [6, 19]. This stream’s natural water supply is augmented by a pisciculture facility some 2–3 km inland, at − 14.32813°, 35.128351°. Here, water is directly taken from the lake and pumped underground, returning aboveground following this stream’s natural path. Before pisciculture, this stream was seasonal but is now a conducive habitat throughout the year, and it is reasonable to speculate that the presence of *P. columella* here was fully or partially attributable to local development(s) in pisciculture along the Lake Malawi shoreline. Another contributing factor would be the introduction and subsequent dispersion of invasive aquatic plants, such as water hyacinth (*Pontederia crassipes* Mart., 1823), now common across all collecting sites, on which *P. columella* was often found.

Following the Upper then Lower Shire River some 200 km southward, *P. columella* was found at Chikwawa 1, a natural and permanent oxbow lake of the Lower Shire River (Fig. 1). Upon more extreme seasonal flooding, this oxbow is directly connected to the river, which recently occurred in March 2023 by cyclone Freddy. Moving a further 100 km southward, both Nsanje 4 and Nansje 5 are seasonally flooded areas for informal pisciculture and small holder rice farming. Each site is temporarily connected to the Lower Shire Valley upon natural inundation(s) and by managed sluice gates (Fig. 1).

Water chemistry data were collected for each snail sampling site at each visit, including data for temperature (°C), pH, conductivity (µS) and total dissolved solids (TDS; ppm). Average values for these four water chemistry parameters at the sampling sites were: (i) Mangochi 1: 27.8 °C, pH 8.1, 517.6 µS and 264.6 ppm, respectively; (ii) Chikwawa 1: 32.7 °C, pH 8.4, 627.2 µS and 308.7 ppm, respectively; (iii) Nsanje 4: 32.5 °C, pH 8.3, 443 µS and 221.8 ppm, respectively; and (iv) Nsanje 5: 30.1 °C, pH: 8.0, 436.3 µS and 202.7 ppm, respectively. Across all 12 sites surveyed and across all time points, the average water chemistry data were 29.3 °C, pH 8.2, 487.9 µS (conductivity) and 244.9 (TDS). These environmental parameters are broadly consistent to those where *P. columella* has been found elsewhere [18] and are typical of predominantly permanent water bodies with a muddy substrate and various vegetation present.

Current information on the epidemiology of liver fluke infection of livestock in Malawi is scant as formal reporting of infection in slaughtered animals has ceased. In the past, however, northern parts of the country carried the greatest burdens while prevalence in Mangochi, Chikwawa and Nsanje Districts was comparatively low [20]. Our observations suggest that these infection trends will not remain the case with a new alien host of liver fluke present.

To summarize, our report of *P. columella* considerably expands the known geographical range of this alien intermediate host snail species across southern Africa. We add to the malacological list of alien freshwater snails in Lake Malawi and Lower Shire River, contributing to a growing list of evidence for wider ecological change. We outline a new and pressing need for further and more thorough surveillance of human and animal fascioliasis in Malawi.

## Data Availability

No datasets were generated or analysed during the current study.
